# Experiences of human papillomavirus self‐sampling by women >60 years old: A qualitative study

**DOI:** 10.1111/hex.13707

**Published:** 2023-01-12

**Authors:** Karin Blomberg, Maria Hälleberg‐Nyman

**Affiliations:** ^1^ School of Health Sciences, Faculty of Medicine and Health Örebro University Örebro Sweden

**Keywords:** cervical screening, HPV, human papillomavirus, qualitative, self‐sampling

## Abstract

**Background:**

Human papillomavirus (HPV) self‐sampling has shown to be acceptable and feasible across cultures and effective in reaching women who do not participate in regular cervical cancer screening. However, most of these studies have included younger women. There is a lack of knowledge of how older women reason about HPV self‐sampling.

**Objective:**

The aim of this study was to describe how women (>60 years old) experience the offering of self‐sampling of HPV, compared to having a sample collected by a healthcare professional.

**Design and Participants:**

The study had a qualitative explorative design. Four focus group discussions were conducted with women 60–69 years old (*n* = 22). Data were analysed using principles of interpretive description.

**Results:**

Five themes were identified: self‐sampling—convenient and without pain, lack of knowledge, worries related to HPV self‐sampling, need for information and taking a societal perspective.

**Conclusion:**

Women aged >60 years found that HPV self‐sampling was convenient and easy to perform. Further, they stressed the importance of being able to remain in the screening programme in advanced age and that self‐sampling could be a possible solution. This study also revealed a lack of knowledge among women in this age group regarding HPV infection, how the disease is transmitted and its relation to cervical cancer.

**Public Contribution:**

Women who had performed HPV self‐sampling participated in the focus group discussion.

## 1 BACKGROUND

Screening as secondary prevention to reduce the risk of cervical cancer has been established for decades, shown to be effective and organized as a population‐based programme.^1^ In the last 10 years, knowledge of the human papillomavirus (HPV) as a necessary prerequisite to cervical cancer has changed the prevention methods from primary cytology testing to analysis of HPV.^2^, ^3^, ^4^ HPV vaccination as primary prevention is now offered in many countries, mainly to young girls before sexual debut as the main target group.^5^ As a complement to the cytological analysis, an HPV test (testing for high‐risk types 16 and 18 that cause 70% of all cervical cancer) is provided in several European countries, as well as in the United States,^6^, ^7^ recommended by the WHO.^8^ HPV testing has even been shown more effective than screening by cytology.^9^, ^10^

Participation in prevention programmes is a key to reducing the incidence of and mortality from cervical cancer. Different barriers to attendance, as well as factors that encourage women to attend the screening, are well‐described in the literature, for example, by our previous studies.^11^, ^12^, ^13^, ^14^ Nonattenders of the cervical cancer screening programme have been described as having an increased risk for cervical cancer.^15^, ^16^ Self‐sampling of HPV has been implemented and has shown to be effective in reaching women not attending the regular screening,^17^, ^18^ as well as being experienced as acceptable and feasible across cultures.^18^, ^19^

Several studies have been conducted evaluating HPV self‐sampling,^20^ but most of the research has included younger women <60 years of age. To exclude older women from studies is problematic: HPV screening may be especially important for this age group, as cytology as a primary screening method has low sensitivity for women >60 years. Additionally, older women are often excluded from regular invitations to screening programmes, and cervical cancer found often is identified late, leading to a more invasive form of cervical cancer.^21^, ^22^, ^23^

This study is part of a project evaluating HPV self‐sampling in women aged 60–69 years compared to having a sample collected by a healthcare professional, with respect to conformity (sensitivity, specificity). In Sweden, HPV testing has been recommended by the National Board of Health and Welfare since 2016 as the primary screening method for women 30 years and older, taken by midwives in the screening programme. Additionally, the upper age limit for screening was increased from 59 to at least 64 years of age, where the woman now is called for her last sampling at 70 years of age.^24^ Larger studies on the prevalence of HPV and how screening works are lacking in the older age group.^25^ At the same time, one‐third of all cervical cancer cases are diagnosed in the age group over 60 years.^21^, ^26^ A large number of studies have been conducted to evaluate self‐sampling with varying results, but studies in an older age group are scarce.^27^, ^28^, ^29^, ^30^

Previous studies have emphasized factors such as information, communication and trust as essential in a well‐functioning screening programme with high coverage.^18^, ^31^ However, knowledge is lacking on how older women >60 years reason about cervical cancer and its prevention. Additionally, there is a lack of studies that include women's own perspectives. Studies evaluating the self‐sampling of HPV are often cross‐sectional or trials^32^, ^33^, ^34^, ^35^, ^36^, ^37^, ^38^; very few qualitative studies exist, but these have either used a theory‐based interview guide that also guided the analysis,^39^, ^40^ or focused on specific groups of women with different ethnicity.^41^, ^42^, ^43^, ^44^ Therefore, the framework for this study is based on naturalistic enquiry^45^ that fosters an inductive approach, meaning that instead of relying on predetermined conceptualizations, aims to generate new knowledge and understanding by interacting with the participants in their own lived experience. The aim of this study was to describe how women >60 years of age experience the offer of self‐sampling of HPV, compared to having a sample collected by a healthcare professional.

## 2 MATERIALS AND METHODS

### 2.1 Design

This qualitative explorative study using focus group discussions (FGDs) was part of a larger study on the evaluation of vaginal self‐sampling of HPV (https://researchweb.org/is/fourol/project/228071 Reg. no 228071) in all women aged 60–69 years in Örebro County in Sweden (*N* = 2258) who were invited to a ‘catch‐up’ screening programme, arranged and funded by the health care during 2018–2020. This catch‐up programme is part of the population‐based cervical screening programme in the county and aims to be a bridge between the earlier screening programme that women exit at 59 years and the ‘catch‐up’ to 70 years. Exclusion criteria were being deregistered from the population‐based screening programme due to prior hysterectomy or own request or having a history of invasive carcinoma. Women were given information about the main study along with the invitation to the ‘catch‐up’ screening programme.

### 2.2 Participants

All women who had given informed consent to the main study on vaginal self‐sampling of HPV were informed about this focus group study. A strategic sample of women included in the main study, based on variations in HPV test results, were asked to take part. FGDs were held in two different strata: women with the same result in the self‐sample and in the midwife‐obtained sample and women with different results in the two tests. The women were contacted by a researcher who explained the study and invited them to participate. If the women agreed to participate, they were sent further written information on the study. A total of 22 women agreed to participate in the four FGDs (two groups in each stratum). The FGDs included five to six participants each.

### 2.3 Data collection

Data were collected in FGDs, a form of group interview that capitalizes on communication between research participants to generate data. Further, it is known that potentially delicate issues are more suitable for group discussions than individual interviews.^46^, ^47^, ^48^ All FGDs took place at the university and were held in Swedish, moderated by the first author (K. B.) and co‐moderated by the last author (M. H.‐N.), both with experience and training in performing FGDs. All FGDs began with the moderator welcoming the participants and providing information on how the discussion would be conducted. The FGDs began with a discussion on the question ‘What is your experience with HPV testing?’ and followed an open interview guide. The FGDs lasted between 35 and 45 min, and all interviews were audio‐recorded. Data collection and the first steps of the analytical process were conducted in parallel, as women's reasoning in prior FGDs was discussed and confirmed in the next one.

### 2.4 Data analysis

The audio recordings of the FGDs were transcribed verbatim by a professional secretary. Data analysis was inspired by interpretive description.^49^, ^50^ First, transcripts were read by two of the researchers separately using constant comparative techniques and involved asking the questions ‘What is going on here?’ and ‘What is this about?’ while reading sections of the text. The researchers identified patterns, generating initial codes inductively. Moderators' notes of reflections during the data collection were taken into consideration in the analysis. The preliminary coding structure was revised through a comparison of the coded transcript excerpts. This was followed by searching for themes, reviewing themes and defining and naming themes.^51^ Second, the emerging themes were interpreted through a ‘clinical lens’, based on professionals' backgrounds as nurses and prior research conducted on cervical cancer screening. Preliminary results were continuously discussed during the analytical process in the larger research group, which included researchers with different professional backgrounds (GP/pathologist, biomedical scientist, registered nurses) and midwives working with cervical cancer screening. For examples of the analysis process, see Table 1. Quotations were identified from the FGDs to illustrate the findings and increase trustworthiness.^45^

**Table 1:** Examples of the analysis process

Meaning unit	Code	Theme
W: It's like less painful or unpleasant to.	Less pain and discomfort	Self‐sampling—convenient and without pain
And especially for those who felt uneasy in the gynaecology chair	Avoid the gynaecology chair	
W: I could take the test when I had time, and when I felt like doing it. I didn't have a time to fit. So that was great.	Be able to control time and place	

## 3 RESULTS

The findings are presented in the five themes formed in the analysis, all related to women's experiences of HPV self‐sampling.

### 3.1 Self‐sampling—Convenient and without pain

Overall, the women described the self‐sampling as easy to perform and found the instructions were clear. Even though most of them were retired from work, they expressed that it was convenient to take the sample at home on an occasion that was suitable for them. Not having to schedule a time and not having to travel were described as advantages of the self‐sampling. When taking the self‐sample at home, they could do it slowly and did not have to hurry. They expressed that they had experiences of feeling rushed and of being on a conveyer belt when visiting the midwife for Pap smear tests and that there was no time for conversations or asking questions.

W*1: When you go to your midwife to take the sample they don't have a lot of time. It is much like a conveyer belt. (FGD 3, *n* = 6)

*W = woman

The women shared experiences of how having a sample taken by the midwife often could be painful. However, they described that the self‐sampling did not hurt, as the sample was collected from the vagina and not from the cervix. They also were relieved that they could take the self‐sample without entering the gynaecological examination chair, which could be more difficult in advanced age and which several of the women described as a vulnerable situation.

W1: It was so easy, so convenient. It was no trouble at all to take the test. Yes, simple you got all the equipment you needed, and the form was already filled out. It was just for us to put the stuff in the envelope and put it in the mailbox.

W2: It was very good, there were good instructions. It wasn't difficult.

W3: No.

W4: At first, I thought like, ‘Oops, how is this going to work?’ But, soon after, I thought that I should be testing. There was nothing strange at all.

W5: No, and you didn't have to ‘climb’ the gyno chair. Because that can feel unpleasant. I have always attended. I've come when I've been summoned. It isn't that fun, but there are things that are worse. (FGD 1, *n* = 5)

### 3.2 Lack of knowledge

It was obvious from the FGDs that the women lacked knowledge about the central aspects of cervical cancer and its prevention. Several women did not know what HPV was or that the HPV test and the Pap smear were not the same tests.

W1: Then I have to ask what it is [HPV], because I have not heard of it?

Interviewer: No, you have not heard of it at all?

Several W: No. (FGD 2, *n* = 5)

Almost all of the women expressed that they had attended regularly at the population‐based cervical cancer screening over their lifetime but never reflected on why they took the Pap smear and the intervals. Several women expressed that they did not know that they would not be offered an invitation to the cervical screening programme, anymore.

W1: Because I was not called, so then I thought, I have to call and ask. ‘No’, she said. ‘You have turned 60 year, you know, so you cannot come anymore’. I thought ‘What?’ (FGD 1, *n* = 5)

It emerged in the FGDs that it was not clear to most of the women that the screening programme had changed over time.

W1: What is it really you look for when you take the test at the midwife's … I did not have knowledge, I think. I go there, and they take that test, and then I trust it. But then there is [information] about this virus … if it exists and then how it has affected? (FGD 2, *n* = 5)

The women described thoughts about how they could take the test by themselves, as they lacked knowledge of the technical procedure of the tests. They reflected on whether the test taken by themselves really was correct, as the test (Pap smear) they had done by the midwife feels and sometimes hurts compared to the self‐test.

W1: I thought … do you have to scratch the cervix or is it [the HPV virus] … everywhere in the vagina I wondered. How do I know that I find the right spot?

W2: I felt the same.

W3: Yes, me too.

W2: Or wondered…

W3: Yes, because when you are at the midwife, you can feel how she almost…

W2: Yes.

W1: Yes, exactly … maybe it is all over the space. I don't know.

W2: No, but that was exactly the same as I thought. Because they say, ‘Oh, it might hurt a bit’. (FGD 1, *n* = 5)

Reflections about whether the tests were the same or not were also especially apparent among those women who received a positive HPV result when the test was done by themselves and a normal result when done by the midwife. They expressed frustrations about the different test results and about not being given any explanation as to why the tests differed.

W1: The test showed that I have the virus.

W2: The same for me.

W1: And I got scared, so I had to call you at the hospital … and got an explanation from—not the person I talked to at first, but from someone else. I got the explanation that the self‐test sample is not performed the same way as the midwife test. The midwife test is from the cervix but the self‐test is just from the area near the cervix. But I got scared, and I think about this almost every day. (FGD 3, *n* = 6)

Some women knew that the HPV test was about the prevention of cervical cancer but expressed little knowledge of the disease itself, such as its causes and possible prevention. Even those women who had experienced cytological abnormality and treatment for that expressed uncertainty about the causes and progression of the disease. They were surprised that they could have gotten the infection many years ago and not have had any symptoms. Some women thought that a positive HPV test was equal to having cancer. Other women described how a positive HPV test led to a fear of developing cervical cancer.

W1: Yes, the information, you know, one goes and wonders, ‘Will I get cervix cancer now?’ (FGD 4, *n* = 6)

For several women, it was unknown that HPV is a sexually transmitted infection. Few women described a connection between HPV and sexual partners and cervical cancer, and some mentioned the HPV vaccination for young girls as a connection. However, most of them were not aware before entering the study that HPV could appear in women in their age group. They discussed HPV being a ‘new’ infection and something that occurred only in teenagers and young people.

W1: We who struggle with the same old man, it is not as important…. (FGD 4, *n* = 6)

W3: In our age it isn't of that great importance [HPV vaccination], if we already caught it [HPV], it doesn't matter where we got it from.

Several women: No, no.

W3: But it is, of course, important information for young persons. (FGD 4, *n* = 6)

### 3.3 Worries related to HPV self‐sampling

The women expressed several different kinds of worries related to the HPV self‐sampling. There were women who described being worried that they had performed the self‐sample in an incorrect way. They were concerned about whether they had rubbed the swab deep enough to collect any virus. There were also worries about whether the self‐sample was as reliable as the sample taken by the midwife. And there were also some women who expressed a worry about other diseases that could be detected by a midwife beside the HPV test that was taken. It was seen as beneficial to have extra control to have it confirmed that one was healthy.

W1: But are there other … diseases or symptoms or … that a midwife can see when you are sitting there that you may also be able to treat? (FGD 1, *n* = 5)

Women in the FGDs who had a positive HPV test also expressed other worries—worries about from whom they had got the HPV virus and worries about being infected with HPV without knowing. Not knowing for how long they had been infected with HPV generated reflective thoughts about how they had lived their lives when they were younger. There were also women who became suspicious towards their partner, wondering whether their partner had cheated on them or where else they could have got the HPV virus.

W1: …then I asked my husband, ‘Have you been with anyone else?’ Because HPV can't be for 30 years, can it?

W2: Well…

W3 & 4: Yes.

W1: Well, the feeling at first, ‘Have you been with anyone else?’ No, well, it does matter. Like that, but otherwise this [the self‐test] is great. (FGD 3, *n* = 6)

W1: Yes, but I know, because I have been cohabiting for 10 years, and it makes you wonder, when did I get it [HPV virus] … A certain fear … My first thought was, ‘Did I get it from you or did I get it before I met you?’ Well, it is not strange to ponder about this.

W2, 3: No, no.

W1: Because you don't ask a man about such things.

W2: No.

W3: No. (FGD 4, *n* = 6)

A positive HPV test also caused worries about having cancer and whether they were going to die. One woman described how she identified different signs in her body that could be related to being ill:

W1: Yes, I was worried, and I must say that I googled, too, and then I experienced and thought that I started to smell strange around me … so then I called actually to the clinic and asked…. (FGD 4, *n* = 6)

They also expressed concerns about whether the information from the doctor and midwife was reliable. There were also descriptions of how seeking information on the Internet could increase their worries. There was an overload of information, and they described information that was in some ways contradictory. It was expressed that it could be difficult to know which websites, besides Sweden's 24‐h helpline 1177, provided reliable information.

W1: …to get an answer [a positive HPV test] and get an understanding of what this answer means, because that's what you may not know … And then maybe you get worried and then you go to the Internet and read, ‘Yes, this is how it can be’, but talk to a person then about ‘I feel this worry. Is it justified or not?’ and get it explained. It is probably the most important thing. (FGD 2, *n* = 5)

### 3.4 Need for information

The women expressed that information was crucial, especially if the HPV self‐sampling was to be implemented for all women. Not having contact with a midwife made the information even more important.

W1: HPV self‐sampling, if it is to become a routine then, then I think they need to think about the information, because now we all have different experiences. Then you meet with no one. (FGD 4, *n* = 6)

In the FGDs, two different needs for information were raised, the need for information regarding how to perform the test, but also a need for information when receiving a positive test result.

W1: Yes, information is so important, because you go and wonder like, ‘Well, will I get cervical cancer now?’ (FGD 4, *n* = 6)

Although, it was appreciated that the information included images on how to perform a test, the women still perceived that they lacked information regarding how to perform the test. They also lacked information that the self‐sample kit was safe and that it was not possible to do it wrong. Also, some women expressed that they would have been helped by information on how to perform the test if one had fragile tissue or vaginal prolapse. These women described that they at first could not perform the self‐sampling and that they had contacted the midwife at their health centre and been advised to use Vaseline or Estriol to facilitate the sampling.

Further, the women expressed that an instructional video on the Internet could be a complement to the written instructions, even though there were illustrations in the instruction booklet. An instructional video could be useful for all women, but especially for women who could not read instructions in Swedish.

It was emphasized that the information regarding how to perform the self‐sampling, when to expect a result and what happens if the result is positive is of extra importance as there is no contact with health care and no opportunity to ask questions.

W1: That you get a phone call, not paper.

Several W: No, no.

W1: It always raises questions, and then you cannot get an answer on a piece of paper.

W2: No, but exactly, I called. You feel that you want to know, why is there a difference, yes.

W4: Yes, yes, I had to come up with such a real examination, but then after that I did not get any information and I missed it. So I was at the clinic here, and they did a real check … It was okay, but you would still have wanted a message afterwards that ‘this is what it looks like or this is what it looks like; it does not come out’. And I still have not received it. It is tedious. (FGD 3, *n* = 6)

### 3.5 Taking a societal perspective

The FGDs revealed that the women felt that they were contributing to society by taking part in this self‐sampling study. All the women had attended the standard screening programme over the years, but they expressed that self‐sampling could be an alternative for women who do not attend the standard screening programme. For example, immigrants or women with negative experiences of gynaecological examinations or abuse might find the self‐sampling acceptable. Further, as well as self‐sampling being seen as a way to increase the participation rate, it was also seen as an option for ongoing testing of women over 60 years of age, as it is not resource intensive.

W1: …to present the arguments for [continuing HPV testing] instead of, as I said before, ‘Well, now, aren't we even allowed to do this [HPV testing], anymore? There are no nurses, and you are not allowed go to the primary healthcare centre, and now shall you do the HPV self‐test as if you were a gynaecologist?’ I can really hear what it would be like if you did not get the information about the positive aspects of self‐testing.

W2: Yes.

W3: Uh‐huh.

W1: And also, I think that we all feel that if we can contribute to reducing the costs, it feels sensible.

W4: Definitely! (FGD 1, *n* = 5)

Self‐sampling was described as a way of freeing up time and resources for the midwives. On the other hand, one woman expressed a concern that self‐sampling could increase societal costs, as more women would participate in the screening programme, and as the HPV self‐sample seems to be more sensitive than the test taken by the midwife, which would lead to increasing numbers of follow‐up examinations.

W1: It's about the information about why and in what way you contribute … Because I mean, just like you are in, if many more go, you can prevent, if you should have cancer or get cancer, that it spreads before you get help, and in the long run, everyone benefits from it. And that healthcare can focus on those who really need help. (FGD 1, *n* = 5)

Most women were aware of the HPV vaccination of young girls, and they thought it was important also to vaccinate young boys. Further, they discussed whether not only women but also men should be tested for HPV. In one of the FGDs, the heteronormative aspect of HPV testing and vaccination was reflected on.

## 4 DISCUSSION

The present study examined how women aged 60–69 years experienced HPV self‐sampling. To our knowledge, this is the first qualitative study on experiences of HPV self‐sampling on women in this age group, compared to having a sample collected by a healthcare professional.

Overall, the women were positive towards self‐sampling and found it convenient and easy compared with going to the midwife. This is in line with results from earlier studies on younger women.^39^ Even though most of the women in our study were retired from work, they still appreciated being able to perform the test whenever they wanted.

The women were familiar with the HPV vaccination among young girls, but they had not reflected on the fact that older women could also be infected with HPV. Further, not all of the women were aware that HPV is a sexually transmitted infection and that it could cause cervical cancer. A knowledge deficit is also reported in younger women in earlier research, where the women also described a lack of knowledge and a need for more education on HPV and cervical cancer.^52^, ^53^ This result is surprising, in that despite those women >60 years having regularly attended screening programmes during the majority of their lives, they lacked knowledge about screening methods and the causes and prevention of cervical cancer. Thus, it highlights that most women participate in screening without knowing why, and there remains a need to find different ways to communicate information so that women could give more informed consent based on evidence. Several of these women had suggestions of how to improve information, for example, through instructional videos, especially important for women who do not speak Swedish. They discussed how it is difficult to navigate and judge different information published on the Internet and how to get women to only read established and evidence‐based websites. In the FGDs, many thoughts were raised about cervical cancer and how it could be detected in time. There may be a need to target just this age group of women with, for example, information about risks for cancer and its prevention, but also more practical information such as how to perform the self‐test with fragile tissue or vaginal prolapse. Prior studies have investigated different interventions, mainly focussed on providing information to younger age groups of women^54^, ^55^; however, it is a methodological challenge to evaluate the effect of such interventions. In this study, no women expressed that they had difficulty understanding how to perform the self‐sampling test. However, several described uncertainties about having done it correctly. Williams et al.^53^ showed that women have low confidence in their ability to take the self‐sampling test correctly. In recent studies comparing the self‐sampling of HPV to having a sample collected by a clinician, the results showed a concordance between the two testing methods.^17^, ^20^, ^56^, ^57^, ^58^ This stresses the need to provide the women with information that the HPV self‐sampling test is equal to having a sample collected by a clinician, and is reliable.

It was obvious in the FGDs that these women expressed a trust that other abnormalities or diseases could be detected during cervical cancer screening but concluded that these could be missed in a self‐sampling HPV test. It might be in some way a ‘false’ trust that it is possible to detect diseases during this short examination/test. In any case, it might be understood in the light of it being more common in this age group to have established contact with a midwife over one's lifetime or to have had regular gynaecological examinations and/or opportunistic screening by a private clinic. It might be different in younger age groups, as much of the behaviour is related to socialization over time in health prevention programmes. This trust in the midwives could also explain why many of these women expressed being disappointed not to be offered regular invitations to screenings, anymore. In the context of being older and having an increased risk for diseases such as cancer, they pointed out that they need to be checked and that the self‐sampling tests could be an alternative. This must, however, be considered along with the increasing risk of ‘false’ positive HPV tests that would need to be followed up and an increasing need for correct information about what a positive HPV test means.

In the light of the Covid‐19 pandemic, it is even more urgent to evaluate and implement HPV self‐sampling, as the conventional HPV screening programme is set on pause to minimize the risk for transmission of infection.

## 5 LIMITATIONS

A potential limitation of this study is that all women in the FGDs had performed the self‐sample as well as having had the sample performed by the midwife. Even though the main study invited all women registered in the ‘catch‐up’ screening programme, there is a risk of a selective sample, not targeting women who were difficult to reach or who had low levels of participation in population‐based screening. We made an effort to arrange a focus group with women who had not performed the self‐sample to capture descriptions of reasons not to perform the self‐sample. These women either declined to participate in a focus group or did not show up for the focus group. This might have led to a more positive description of the self‐sample, even though the included women did describe the disadvantages as well as the advantages of self‐sampling. Future research focusing on women who are hesitant to use HPV self‐sampling is needed to get more complete knowledge.

## 6 CONCLUSION

Women >60 years of age found that HPV self‐sampling was convenient and easy to perform. Further, they stressed the importance of being able to remain in the screening programme in advanced age and that self‐sampling could be a possible solution. This study also revealed a lack of knowledge among women in this age group regarding HPV infection, how the disease is transmitted and its relation to cervical cancer. Information targeting just this age group of women is needed, along with practical instructions on how to perform the test with fragile tissue or vaginal prolapse.

## CONFLICT OF INTEREST

The authors declare no conflict of interest.

## ETHICS STATEMENT

Ethical approval was obtained from the Regional Ethical Review Board of Uppsala, Sweden (reg. no: 2017/365).

## Data Availability

The data are not publicly available due to privacy or ethical restrictions.
